# Topography is a Major Determinant of Forest–Savanna Distributions in Mosaic Landscapes in Central Africa

**DOI:** 10.1007/s10021-026-01070-2

**Published:** 2026-05-27

**Authors:** Aart Zwaan, Arie Staal, Mariska te Beest, Max Rietkerk

**Affiliations:** 1 Copernicus Institute of Sustainable Development, Utrecht University, Utrecht, The Netherlands; 2 Centre for African Conservation Ecology, Nelson Mandela University, Gqeberha, South Africa

**Keywords:** alternative ecosystem states, bistability, ecotone, feedbacks, forest–savanna mosaics, tipping points, topography, tropical ecosystems

## Abstract

Forests and savannas frequently co-occur as patches within tropical landscape mosaics, yet the mechanisms controlling their spatial configuration remain unclear. The presence of both vegetation states under similar climatic conditions is often attributed to fire–vegetation feedbacks, but could also reflect variation in overlooked external drivers. In Central Africa, forest–savanna mosaics become more common with increasing topographic roughness, but how well topographic heterogeneity explains the forest–savanna configuration within mosaic landscapes is unknown. Here we address this question and examine the role of individual topographic variables that may influence tree cover by, for instance, changing water availability and fire spread. We identify mosaic landscapes from remotely sensed tree cover data and derive topographic variables from a digital elevation model. We use these variables to develop machine learning algorithms predicting vegetation state within mosaic landscapes. Models achieved an average prediction accuracy of 0.75, with local elevation (relative to the surrounding 500 m or 5000 m) emerging as the strongest predictor of vegetation state. Both model accuracy and the role of topographic predictors varied strongly among landscapes, reflecting the diverse pathways by which topography can influence tree cover. Overall, our findings indicate that topographic heterogeneity is a major driver of forest–savanna mosaics in Central Africa. Mosaic landscapes are more deterministic than previously assumed, suggesting that their response to disturbances and climate change will be spatially heterogeneous, thereby reducing the likelihood of abrupt large-scale shifts between forest and savanna states.

**Supplementary Information:**

The online version contains supplementary material available at 10.1007/s10021-026-01070-2.

## Highlights

- Topographic heterogeneity is a major driver of forest–savanna mosaics.
- Forest–savanna mosaics are more deterministic than previously assumed.
- Local elevation is the main determinant of vegetation state.

## Introduction

Over large areas of the tropics, forest and savanna locally co-occur as patches within landscape mosaics (Bernardino and others, 2022; Pletcher and others, 2022; Zwaan and others, 2024). Forest patches are characterized by closed tree cover, whereas savanna patches consist of a continuous grass layer with open tree cover. These two distinct ecosystems are typically separated by sharp boundaries, with intermediate tree cover being rare (van Nes and others, 2018; Cardoso and others, 2021). Their co-occurrence under similar environmental conditions has led to the hypothesis that, within a range of climatic regimes, forests and savannas may represent alternative ecosystem states determined by historical conditions and stabilized by positive feedbacks between vegetation and fire (Hirota and others, 2011; Staver and otherrs, 2011a). This has prompted the classification of large parts of the tropics as bistable ecosystems, which may be susceptible to critical transitions in response to disturbances or gradual changes in external conditions (Bond 2005; Hirota and others, 2011; Staver and others, 2011b; Aleman and others, 2020).

In Central Africa, however, recent evidence suggests that the spatial extent of such alternative states is limited to forest and savanna patches smaller than 10 × 10 km within mosaic landscapes (Zwaan and others, 2024). At larger scales, the distribution of homogeneous forest and savanna landscapes appears to be fully constrained by climatic and edaphic factors. Consequently, critical transitions between forest and savanna could be restricted to the tipping of smaller patches within mosaics, whereas transitions of larger continuous forest or savanna regions will progress via intermediate mosaic states, appearing gradual on broader spatial scales (Zwaan and others, 2024). Mosaic landscapes may thus play a buffering role against changing climatic conditions, providing intermediate states between continuous forest and savanna, harbouring species from both ecosystems (Huntley 2023).

Considering the role that forest–savanna mosaics may play in modulating the response of tropical ecosystems to disturbances and environmental change, it is important to understand the underlying mechanisms controlling their formation and stability. While the drivers of large-scale forest–savanna distributions have been extensively studied (e.g. Lehmann and others, 2011; Staver and others, 2011b; Murphy and Bowman 2012; Williamson and others, 2024), local dynamics within mosaic landscapes remain poorly understood. In these landscapes, climate cannot account for the presence of adjacent open and closed tree cover patches, as it can be assumed to be relatively constant over small distances (Favier and others, 2012). Instead, local forest–savanna co-occurrence can be explained either by positive feedbacks or by environmental heterogeneity (Murphy and Bowman 2012). Positive feedbacks can reinforce existing vegetation states through interactions among vegetation, fire and shading, allowing forest and savanna patches to persist side by side (Hoffmann and others, 2012; Charles-Dominique and others, 2018; Sagang and others, 2024). However, in the African tropics, these feedbacks appear to be spatially constrained, as bistability is not found at larger spatial scales (Zwaan and others, 2024). Alternatively, environmental heterogeneity at local scales can also lead to the formation of patches, for example by changing water availability and fire regimes (Beckett and Bond 2019; Cure and others, 2023). If such heterogeneity is not accounted for, the resulting pattern may be misinterpreted as bistability of forest and savanna under uniform conditions (Higgins and others, 2024).

Topography can be an important source of heterogeneity, affecting forest and savanna suitability by driving local variations in water stress (drought and waterlogging), fire spread, soil characteristics, temperature and light availability (Wood and others, 2011; Moeslund and others, 2013; Cardoso and others, 2023; Mattos and others, 2023). Higgins and others, (2023), for example, speculate that topographic effects could explain more than half of the mispredicted forest and savanna samples in their study. Some case studies have explored the role of topography in more detail, but these are often geographically limited, restricting the generalizability of their findings across broader regions (e.g. Rossetti and others, 2010; Vaughn and others, 2015; Jucker and others, 2018; Beckett and Bond 2019). In Hluhluwe–iMfolozi Park in South Africa, for example, Beckett and Bond (2019) found that forest vegetation persists in areas that are topographically sheltered from fires, but it is not clear whether their conclusions can be extended to other regions, as the relationship between vegetation and topography may vary depending on the environmental conditions (Moeslund and others, 2013). In contrast, studies regarding the drivers of tropical forest and savanna ecosystems conducted at larger spatial scales frequently lack topographic variables (e.g. Hirota and others, 2011; Staver and others, 2011b), or include only coarse measures such as topographic roughness or elevation (Archibald and others, 2009; Lehmann and others, 2011). This can make it difficult to disentangle the effects of fine-scale topographic variation from those of large-scale elevational gradients that correlate with climatic or edaphic patterns. As a result, our understanding of whether forest–savanna mosaics can emerge under any environmental template, or depend on specific topographic settings, remains limited.

The African tropics have been widely studied in the context of alternative ecosystem states and forest–savanna dynamics, offering a well-established foundation for advancing our understanding of biome distributions (e.g. Sankaran and others, 2008; Staver and others, 2011a; Aleman and others, 2020; Higgins and others, 2023). The broad transitional zones in this region, both to the north and south of the Congolian Rainforest, provide a suitable study area to explore the drivers of local forest–savanna co-occurrence. In this study, we aim to assess the extent to which topographic heterogeneity determines the spatial configuration of forest and savanna patches within mosaic landscapes in Central Africa. We combine remotely sensed tree cover data with topographic variables derived from a digital elevation model to develop statistical models that predict vegetation state as a function of topography within identified mosaic landscapes. We evaluate the accuracy of models, identify the most important topographic variables, examine their effects on tree cover, and assess whether these relationships are general or context-dependent.

## Methods

### Scope

The study area encompasses a region in Central Africa (10°N–10°S, 8°E–32°E), identical to that defined in Zwaan and others, (2024). The region experiences high mean annual precipitation and low seasonality near the equator, with decreasing precipitation and increasing seasonality toward the outer regions of the study area (Nicholson 2022). At its centre lies the flat, low-lying Congo Basin. Elevation increases along the basin's margins, with significant orographic zones in the northwest (Cameroon Highlands), east (Albertine Rift) and south (Batéké Plateaux and Angolan Highlands). Moist tropical forest of the Congolian rainforest is found near the equator, while a transition to tropical savanna occurs both to the north and south (Huntley 2023). The transition zone is characterized by extensive areas where forest and savanna locally co-occur within the landscape (Zwaan and others, 2024). This study focuses specifically on these mosaic landscapes.

### Data

The selection of areas with forest–savanna mosaics followed the procedure described in Zwaan and others, (2024). Tree cover data were extracted from the Global Forest Change (GFC) ver. 1.11 (2000—2023) product, which provides high-resolution (0.00025°, approx. 30 m) maps of per cent tree cover for the year 2000 (Hansen and others, 2013). Pixels with extensive human impact based on the Global Land Cover 2000 (GLC2000) product (Bartholomé and Belward 2005), as well as water bodies, were masked out. Remaining pixels were then classified as either closed or open tree cover based on a 65% tree cover threshold. This threshold corresponds to the minimum between the two modes in the bimodal frequency distribution of tree cover across the study area (Zwaan and others, 2024). Forest and savanna were therefore distinguished based solely on vegetation structure. While this structural approach does not capture differences in the floristic composition of similarly structured vegetation (Veldman 2016), it is an effective method for identifying patterns in the distribution of these two contrasting biomes (Hirota and others, 2011; Staver, Archibald, and S.A. Levin 2011; Aleman and Staver 2018). Agreement (open = savanna, closed = forest) between GFC-derived tree cover classes and forest and savanna sites from Aleman and others, (2020) was high (Figure S1.1 in Supporting Information Appendix S1), indicating consistency between structural and field-based biome classifications.

To identify forest–savanna mosaics, the study area was divided into ‘landscapes’ of 0.1° × 0.1° (approx. 10 × 10 km), which were classified based on the fractional cover of open and closed tree cover pixels. Aggregating tree cover in this manner retains information about the structural composition of each landscape, which would be lost if average tree cover was used. Landscapes were considered mosaics when the fractions of both open and closed cover fell between 0.05 and 0.95, ensuring that each mosaic landscape contained at least 5% of both forest and savanna. Landscapes with less than 75% valid (non-masked) pixels were excluded from the analysis. Climatic data on mean annual precipitation and precipitation seasonality were extracted from the WorldClim ver. 2.1 dataset (Fick and Hijmans 2017). The data were aggregated from the original resolution of 1 km to the coarser landscape scale of 0.1° by taking the mean. These variables were then used to explore whether the relative proportions of forest and savanna in mosaic landscapes remain dependent on climatic conditions, as observed in the large-scale, climate-driven distribution of these biomes (Murphy and Bowman 2012).

To investigate whether the local configuration of open and closed tree cover within mosaic landscapes can be explained by topographic heterogeneity, elevation data were obtained from the FABDEM ver. 1.2 dataset (Hawker and others, 2022). This dataset provides surface height at 1 arcsecond (approx. 30 m) resolution, with buildings and tree cover removed. Vertical errors in forests are relatively small (2.88 m) compared to elevation differences within landscapes (Hawker and others, 2022). Elevation data were resampled to match the resolution of the tree cover pixels. The elevation data were first used to generate example landscapes where correlation between tree cover and elevation was strongly negative, positive or absent, to illustrate the varying roles that topography can play. For further analysis, multiple topographic features were calculated from the elevation data. Slope and aspect were calculated using the GRASS GIS plugin in QGIS (Neteler and others, 2012). Aspect values were subsequently transformed into sine (aspect_sin) and cosine (aspect_cos) components for use in statistical modelling. Local elevation was expressed as the topographic position index within a 50 m (TPI_50m), 500 m (TPI_500m), or 5000 m (TPI_5000m) radius. It was calculated using the uniform_filter function from the SciPY python package (Virtanen and others, 2020). The topographic position index allows us to express the relative elevation of a pixels compared to its environment at various distances. This set of variables was chosen to capture the relevant topographic features that could affect the local tree cover configuration, while limiting overlap among them.

### Analysis

To analyse the extent to which topography can explain the local co-occurrence of open and closed tree cover in a landscape, we first trained a single ‘regional’ random forest model using samples from across all mosaic landscapes in the study area. The model predicts tree cover (open / closed) based on the six topographic features described previously. Random forest models are a flexible tool well-suited for exploring interactions that may be nonlinear (Couronné and others, 2018), and they can be used without making assumptions about response curves. We randomly sampled 5 open and 5 closed pixels from each mosaic landscape, ensuring the model focused on predicting local vegetation patterns rather than composition differences among landscapes, which may be driven by climatic factors. While stratified sampling may not produce model outcomes that directly translate to real-world predictions, it can enhance comparability between classes (Ahmed, 2024) and thus provide a useful means of assessing how well open and closed tree cover can be distinguished based on topographic variables. The total number of samples, approximately 100,000, were split into training (80%) and test (20%) datasets. The model consisted of 100 decision trees, with a minimum of 10 samples per leaf and a maximum depth of 10.

Model performance was evaluated using the accuracy score, expressing the fraction of the total predictions that was correct. Impurity-based feature importance was used to identify which topographic features were most useful for predicting cover type. Accumulated Local Effect (ALE) plots were used to quantify the effect of a feature on the likelihood of finding open or closed tree cover. The goal of the regional model was to explore whether general topographic effects could be identified that applied across the whole study area. However, training a regional model with pixels sampled from all mosaic landscapes does not account for the fact that topography–tree cover relationships can vary strongly between landscapes (Moeslund and others, 2013). As a result of this variation, meaningful effects might cancel out when averaged across the study area. To begin exploring how landscape characteristics may influence these relationships, we split the data into three subsets (mostly open / balanced / mostly closed landscapes) and evaluated model accuracy, feature importance, and ALE plots for each. This provides an initial look at how the role of topography in shaping vegetation patterns may depend on overall landscape composition and, indirectly, on climatic context.

To address the potential limitations of the regional model more systematically, we also trained random forest models on a landscape-by-landscape basis. Because the focus of this study is on the distribution of tree cover within landscapes rather than on differences among them, modelling individual landscapes is appropriate. The advantage of training local models is that the tree cover configuration would not be explained by climatic conditions, as these can be assumed constant within a landscape (Zwaan and others, 2024). It also allows us to explore how the influence of topographic features differs across landscapes, depending on their location, composition (the relative proportions of open and closed tree cover) and topographic roughness (quantified as the standard deviation of elevation within a landscape). For each local model, 5000 samples were taken, evenly split between open and closed pixels. Other model settings and evaluation metrics were kept identical to those of the regional model. Because it is impractical to visualize thousands of ALE plots (one for each landscape), effects were summarized by their direction and monotonicity. These values, along with accuracy and feature importance, were mapped and compared against landscape composition and topographic roughness to identify spatial patterns and relationships with landscape characteristics.

## Results

**Figure 1:**
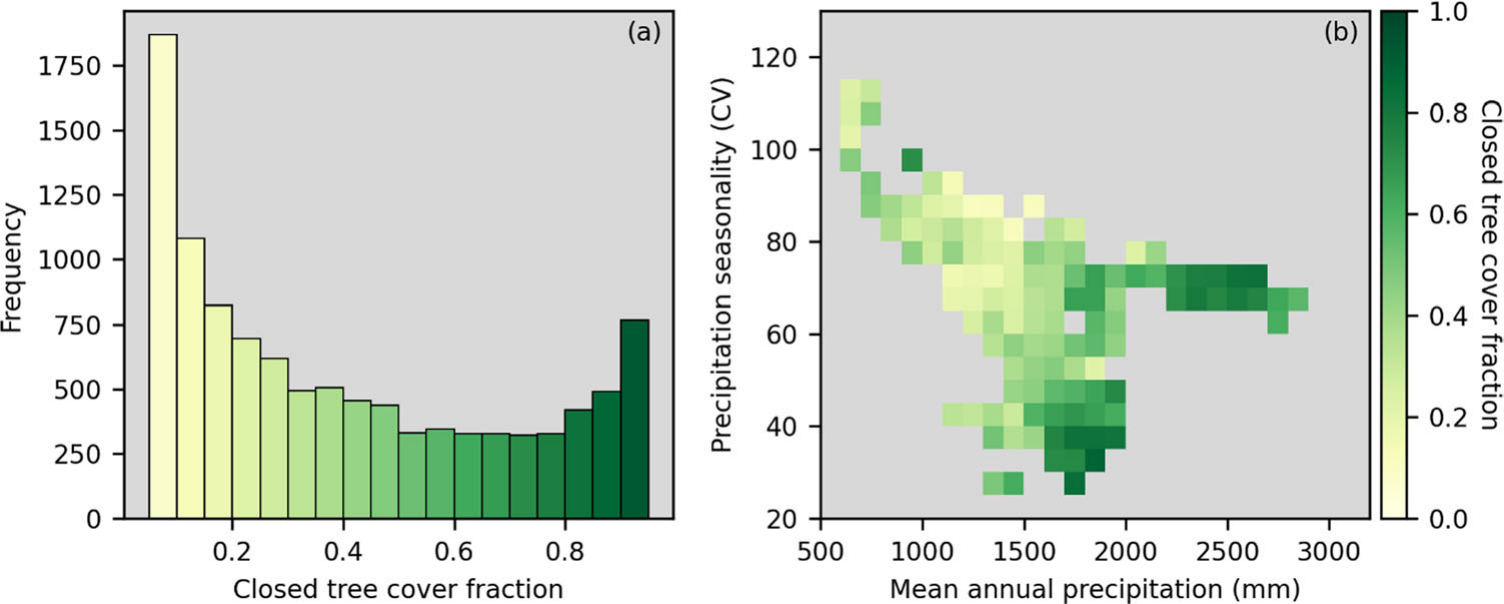
(**a**) Histogram of closed tree cover fraction across mosaic landscapes in Central Africa. Mosaic landscapes are defined as 0.1° × 0.1° areas containing at least 5% open and 5% closed tree cover. (**b**) Heat map of closed tree cover fraction across mosaic landscapes as a function of mean annual precipitation and precipitation seasonality. Only bins containing at least 10 landscapes are shown.

A total of 10,618 landscapes were classified as mosaic landscapes, representing 28% of the landscapes in the study area with natural vegetation and covering over 1.3 million km^2^. Although all these landscapes contained a mixture of open and closed tree cover, the relative proportions varied substantially (Figure 1a). Landscapes dominated by open cover with some patches of closed cover were most common, whereas landscapes that were predominantly closed, or more evenly balanced between open and closed cover, were less common. Mosaic landscapes with more open cover had lower mean annual precipitation and higher precipitation seasonality than those with predominantly closed cover (Figure 1b).

**Figure 2:**
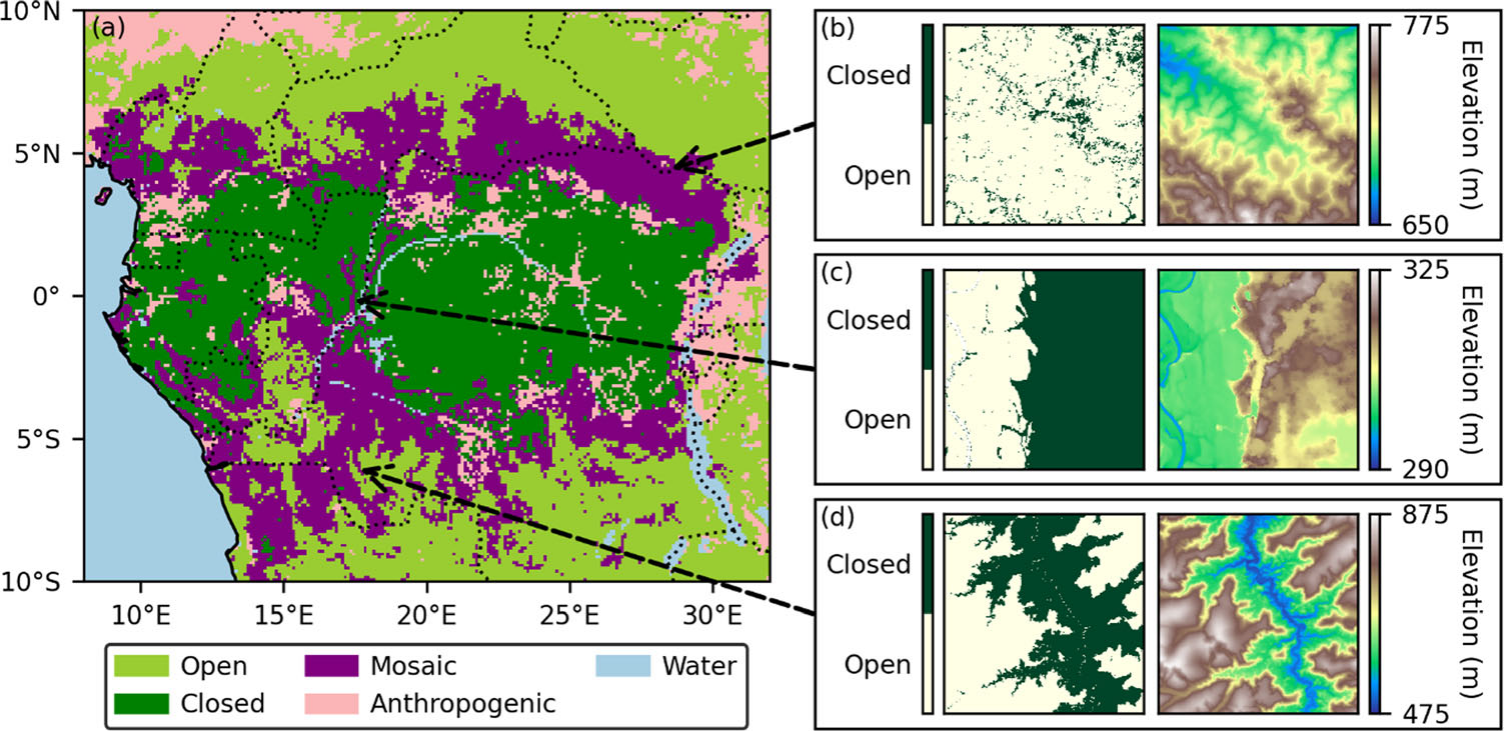
(**a**) Distribution of homogeneous open (> 95% open pixels), homogeneous closed (> 95% closed pixels) and mosaic (≥ 5% open and ≥ 5% closed pixels) 0.1° × 0.1° landscapes in Central Africa. (b—d) Three mosaic landscapes illustrating a lack of (**b**), positive (**c**) and negative (**d**) relationship between closed tree cover and elevation.

We present three distinct landscapes as illustrative examples of forest–savanna mosaics in relation to topography (Figure 2). The accompanying map (Figure 2a) shows the locations of these three landscapes and highlights the broad zones of forest–savanna mosaics both north and south of the Congolian rainforest. Figure 2b depicts a landscape with no clear relationship between tree cover and elevation. In Figure 2c, closed tree cover is located at higher elevations, while open tree cover is found near the river bed. Figure 2d shows a reverse pattern, with closed cover concentrated in low-lying valleys. Elevation differences in this landscape are substantially greater than in Figure 2c.

The regional model, which quantified the average effects of topography on tree cover across mosaic landscapes, had a predictive accuracy of 0.63 (for comparison, a random or null model would be expected to achieve an accuracy of 0.5). Among the topographic variables, TPI_50m was identified as the most important feature, followed by TPI_500m and TPI_5000m (Figure S1.2). The directions of the effects of topographic features showed negative associations of TPI_500m and TPI_5000m with closed tree cover, while a larger magnitude of both negative and positive TPI_50m values was associated with increased closed cover (Figure S1.3). Closed cover generally also increased with slope, although an additional peak was observed at near-flat locations. These findings remained consistent when the data were split into three subsets (mostly open, balanced, and mostly closed landscapes).

**Figure 3:**
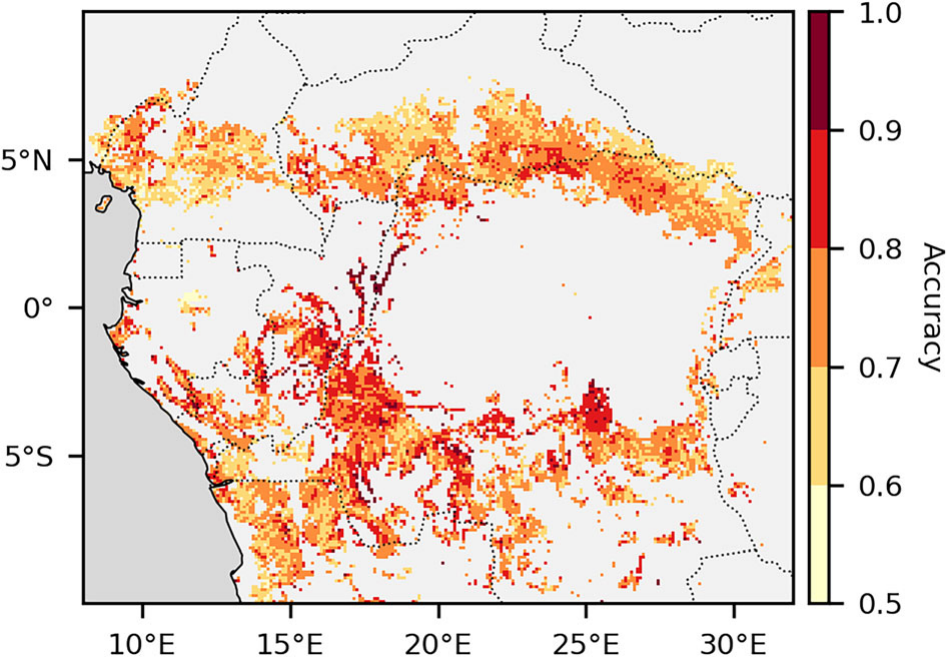
Accuracy of local random forest models predicting pixel type (open vs. closed tree cover) based on topographic variables. Local models were trained and evaluated separately for each 0.1° × 0.1° mosaic landscape. Accuracy is expressed as the proportion of model predictions that were correct. The average accuracy across landscapes was 0.75.

The prediction of tree cover (open vs. closed) in local landscape-by-landscape models, based on topographic features, achieved an average accuracy of 0.75 across landscapes (Figure 3). Accuracy exceeded 0.625 in 95% of landscapes, corresponding to performance at least 25% better than random classification (accuracy = 0.5). In some landscapes, model accuracy exceeded 0.9, meaning that open and closed tree cover could be distinguished with over 90% accuracy based solely on topographic features. High accuracy was concentrated in the centre of the Congo Basin and areas to its south. The lowest accuracies were found in the west of the Congo Basin and along the northern margins of the mosaic zone. Accuracy was not related to the relative proportions of open and closed tree cover in a landscape (Figure S1.4a). However, it was noticeably higher in landscapes characterized by very low topographic roughness (Figure S1.4b).

**Figure 4:**
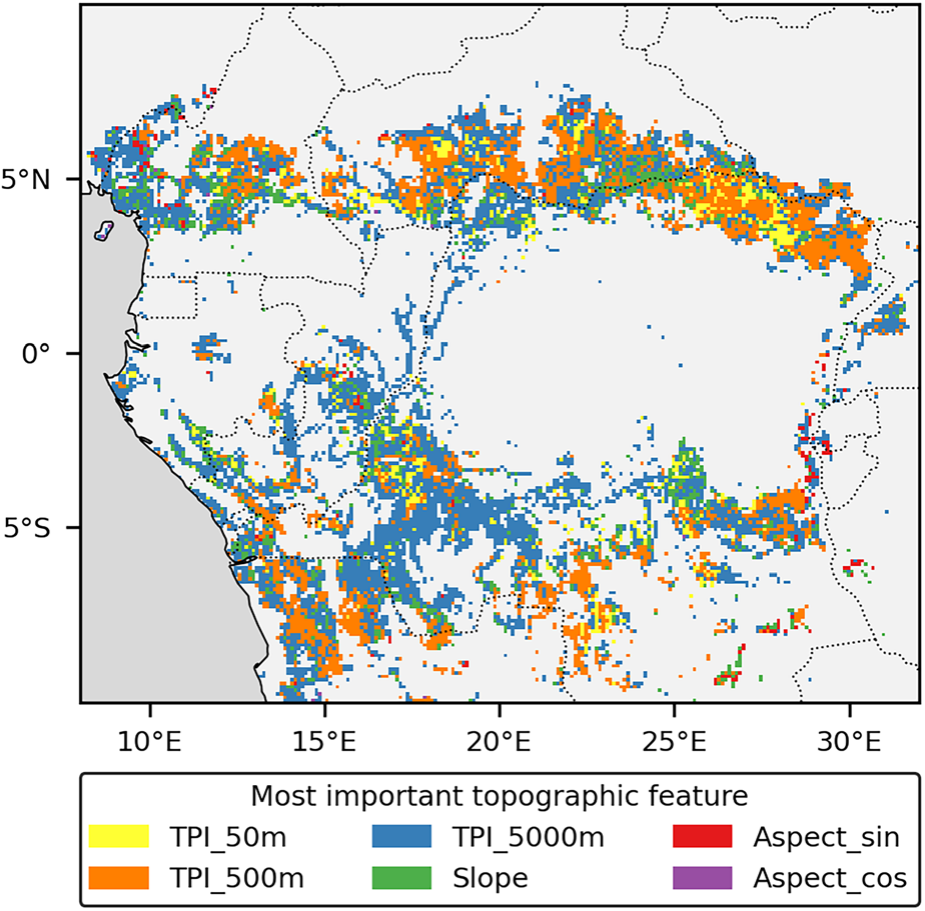
Map showing the topographic feature with the highest feature importance for each local model predicting tree cover based on topographic variables. Local models were trained and evaluated separately for each 0.1° × 0.1° mosaic landscape. TPI_50m, TPI_500m, and TPI_5000 represent the topographic position index (local elevation) relative to neighbourhoods of 50, 500, and 5000 m. Slope indicates the slope angle, while Aspect_sin and Aspect_cos represent the sine and cosine of the slope aspect, corresponding to the eastness and northness of the slope orientation.

TPI_5000m and TPI_500m were most frequently identified as the most important feature in a local model, followed by TPI_50m and slope, which were occasionally the top predictor (Figure 4). These four features also had the highest average importance across all landscapes (Figure S1.5). In contrast, aspect_sin and aspect_cos consistently showed low importance. Feature importance displayed spatial clustering, with the same feature often dominating in adjacent landscapes. Nevertheless, it is difficult to delineate specific geographic regions where individual features dominate, as all features (except aspect) were important in landscapes throughout the entire study area. Feature importance showed some dependence on landscape composition and topographic roughness, with TPI_500m being more important in relatively open mosaic landscapes (Figure S1.6a), whereas TPI_5000m was particularly dominant in the flattest landscapes (Figure S1.6b).

**Figure 5:**
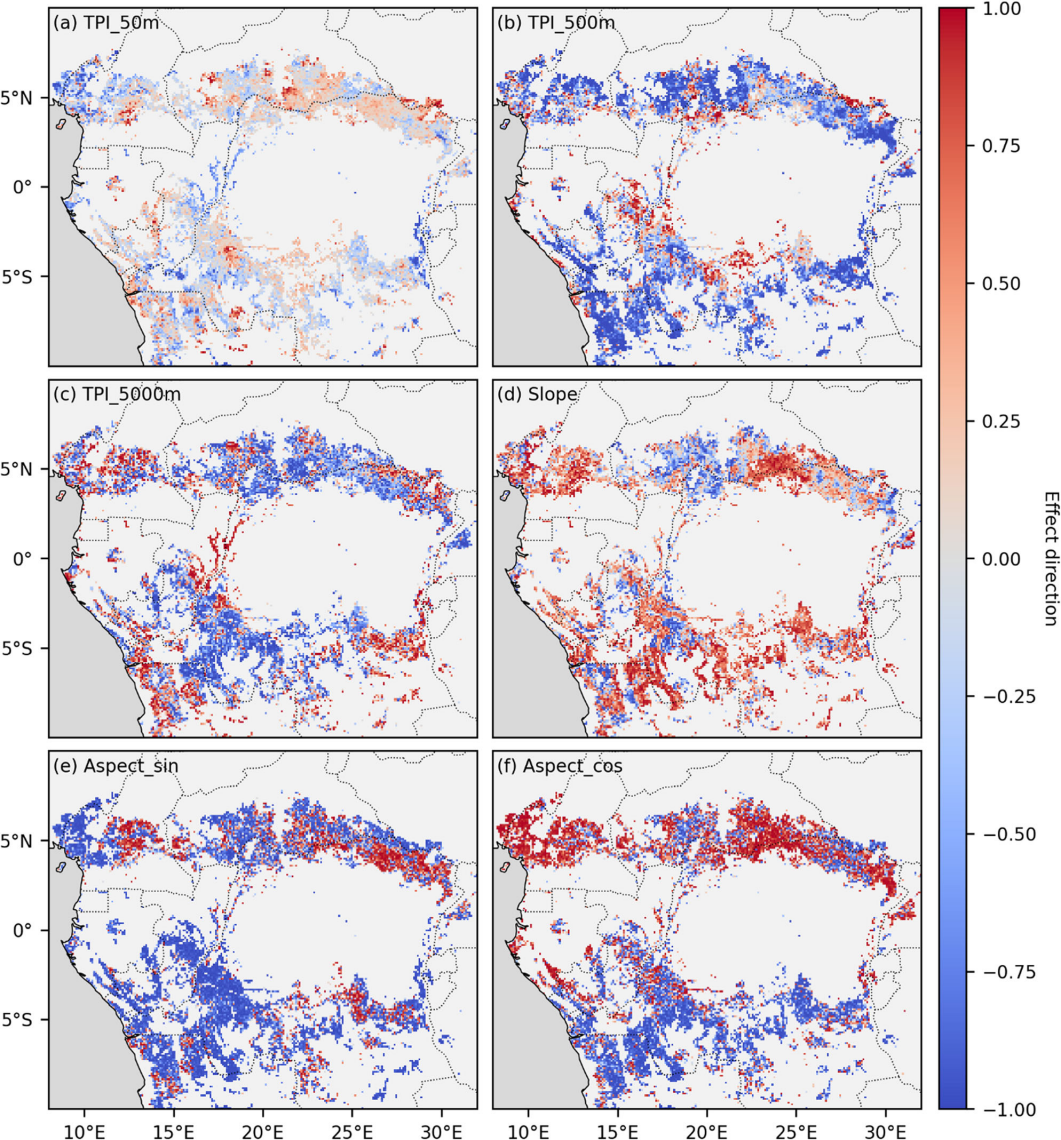
Effect direction of topographic features on the likelihood of a local model predicting closed tree cover, quantified as the Spearman correlation of the Accumulated Local Effect (ALE) plot for each feature. Positive values indicate that higher feature values increase the likelihood of predicting closed tree cover, while negative values indicate that higher feature values decrease this likelihood. The magnitude reflects the monotonicity of this trend within a landscape. Local random forest models predicting pixel type (open vs. closed tree cover) based on topographic variables were trained and evaluated separately for each 0.1° × 0.1° mosaic landscape. TPI_50m, TPI_500m, and TPI_5000 represent the topographic position index (local elevation) relative to neighbourhoods of 50, 500, and 5000 m. Slope indicates the slope angle, while Aspect_sin and Aspect_cos represent the sine and cosine of the slope aspect, corresponding to the eastness and northness of the slope orientation.

The effect direction of features on the prediction of tree cover is visualized in Figure 5. The maps show the correlation between a feature and the accumulated local effect of that feature on tree cover. For each landscape the colour indicates the monotonicity and direction of the relationship between a feature and the likelihood of a model predicting closed tree cover. A negative relationship (blue) indicates landscapes where closed tree cover was more likely at lower feature values. A positive relationship indicates the opposite. The direction of the effect of TPI_5000m on tree cover varied strongly across the mosaic landscapes. It was positive in the centre of the Congo Basin, as well as in the southwestern, southeastern, and parts of the northern landscapes. In contrast, TPI_5000m had a negative effect on tree cover in the central southern and northern regions. The effect of TPI_500m was more consistently negative, with the exception of a few areas around the centre of the study area and in the far northeast. Slope generally had a positive effect on tree cover. TPI_50m had low correlations, indicating unclear effect directions. However, the absolute value of TPI_50m had a strong positive effect on the likelihood of closed tree cover (Figure S1.7). Despite the low feature importance of aspect_sin and aspect_cos, their effect directions showed a clear pattern with higher likelihood of closed tree cover on north-facing slopes north of the equator and on south-facing slopes south of the equator. Closed tree cover was also consistently found more on east- or west-facing slopes within landscapes, with the direction of this relationship regionally varying. The effect direction of TPI_500m was on average more positive in landscapes with a higher closed tree cover fraction (Figure S1.8a). The same was true for slope and less strongly for TPI_5000m. TPI_500m also had a more positive effect on closed tree cover in landscapes with a lower topographic roughness (Figure S1.8b). In landscapes where model accuracy was very high (> 0.9), TPI_5000m showed a strong positive relationship with tree cover and consistently emerged as the most important feature (Figure S1.9).

## Discussion

Collectively, our results highlight the diverse yet important role of topography in shaping forest–savanna mosaics in Central Africa, and the challenges of capturing its locally specific effects over larger areas. In 95% of the mosaic landscapes, topographic variables explained at least 25% of the forest–savanna distribution. However, a regional model using data from across the entire study area performed markedly worse than local landscape models, suggesting that the influence of topography on tree cover is difficult to generalize using these variables. Among the topographic variables, local elevation relative to the surrounding terrain was most important, followed by slope. Forest cover was generally associated with low local elevations, high relief and steeper slopes. However, substantial regional differences were observed, with forests instead favouring higher elevations in some landscapes. Part of this variation could be attributed to differences in landscape composition, and by extension climatic conditions, which may modulate the mechanisms through which topography affects vegetation.

Co-occurrence of forest and savanna under similar climatic conditions is frequently attributed to positive feedbacks between fire and vegetation that can lead to bistability (Staver and Levin 2012; Dantas and others, 2016; Wuyts and Sieber 2023). However, recent evidence suggests that such bistability may be more limited than previously assumed, as forest and savanna patches that appear bistable may instead be shaped by local environmental heterogeneity (Higgins and others, 2023, 2024; Williamson and others, 2024; Zwaan and others, 2024). Our analysis of topography as a driver of the forest–savanna patch configuration within mosaic landscapes provides new insight into the relative importance of feedbacks and heterogeneity. If mosaics were driven purely by feedbacks, the distribution of open versus closed tree cover should be independent of topographic variation, and topography-based models would perform no better than chance (accuracy = 0.5). In contrast, our local models, predicting tree cover based on topographic variables, achieved accuracies consistently above this threshold, averaging 0.75. This indicates that half of the forest–savanna co-occurrence under similar climatic conditions can be explained by local topographic variation rather than feedback-driven bistability. Nonetheless, remaining prediction errors in our models imply that bistability cannot be excluded, although some misclassifications may instead reflect heterogeneity in variables not included in our analysis. Furthermore, the presence of sharp forest–savanna boundaries in areas where topography varies only gradually suggests that mosaics likely arise from the combined effects of topography and positive feedbacks (Favier and others, 2012). Such feedbacks can amplify structural contrasts between topographically determined vegetation states as well as induce true bistability. Further research could explore how environmental heterogeneity interacts with fire-vegetation feedbacks to shape forest–savanna mosaics, extending recent work showing that the strength of these feedbacks is modulated by soil texture (Sagang and others, 2024).

Topography-driven mosaics can buffer ecosystems against large-scale tipping events. In response to changing conditions, local patches may shift between states, but transitions will appear more gradual when viewed at broader scales (van Nes and Scheffer 2005; Bastiaansen and others, 2022; Dearing and others, 2025). Similar dynamics were demonstrated by Higgins and Scheiter (2012), who showed with process-based models that heterogeneity in environmental drivers leads to asynchronous local shifts between forest and savanna, resulting in gradual continent-scale transitions. Although feedbacks likely also contribute to shaping mosaic landscapes, their interaction with topography appears to constrain the spatial extent of bistability and associated tipping points. At spatial scales of 10 × 10 km and larger, homogeneous forest and savanna landscapes are not observed as alternative ecosystem states (Zwaan and others, 2024). Palaeoecological evidence further supports the stabilizing role of mosaic landscapes, with fragmentation helping species persist during climatic fluctuations (Hardy and others, 2013; Huntley 2023). Accurately assessing ecosystem stability and predicting future transitions therefore requires a clear understanding of topography as a driver of mosaics. The regionally variable effects of topography on tree cover imply that generalizations are difficult, and that transitions will depend strongly on locally specific conditions, underscoring the need to consider them in studies of forest–savanna distributions.

Topography influences tree cover primarily by altering water availability and fire spread (Moeslund and others, 2013; Meddens and others, 2018). Although both mechanisms are frequently cited (e.g. Wood and others, 2011; Villalobos-Vega and others, 2014; Beckett and Bond 2019; Mattos and others, 2023), their relative importance across larger spatial scales remains unclear, as they are rarely examined together. The strong but spatially variable influence of topography observed in our study suggests that the importance of fire- and water-related mechanisms varies among regions. The most consistent trend we found was increased tree cover at both higher and lower local elevations relative to a surrounding 50 m radius, supporting previous findings that high local relief can promote closed tree cover by impeding fire spread (Archibald and others, 2009; Lehmann and others, 2011; Ondei and others, 2017). However, the small elevation differences detected at this scale may be susceptible to vertical errors in the DEM data. In many landscapes, closed tree cover was concentrated in local depressions within a 500 m or 5 km radius. This pattern may reflect the role of fire refugia, areas less vulnerable to fires due to their low topographic position (Meddens and others, 2018). Depressions, however, not only limit fire spread but also tend to increase water availability, providing an alternative explanation for the local prevalence of closed tree cover (Murphy and Bowman 2012; Elias and others, 2019; Mattos and others, 2023). Conversely, in other landscapes, closed tree cover was predominantly located at higher local elevations. This positive relationship between local elevation and tree cover was more common in landscapes mostly composed of closed tree cover, corresponding to landscapes with high mean annual precipitation and low precipitation seasonality. In these landscapes, waterlogging in seasonally inundated valleys may prevent tree establishment and increase tree vulnerability to fire (Villalobos-Vega and others, 2014; Flores and others, 2017; Mattos and others, 2023). While topography can also affect tree cover through factors such as wind exposure or light availability (Moeslund and others, 2013), these appear less important in our study. Nonetheless, we observed that closed tree cover was more frequent on slopes receiving less direct sunlight.

The role of topography in shaping mosaic landscapes varied strongly across Central Africa, as reflected by spatial differences in model accuracies, feature importance values, and effect directions. Such variability is expected, since topographic characteristics vary across space and their effect on tree cover may depend on climatic conditions. For example, Zwaan and others, (2024) showed that topographic roughness increases the likelihood of forest–savanna mosaics in drier regions, whereas in wetter landscapes mosaics occur even at very low levels of roughness. These regional contrasts complicate attempts to generalize the role of topography in determining tree cover and may help explain why it is frequently overlooked, despite our finding that topography is a key driver of the local forest–savanna distribution within mosaic landscapes. Developing a generalized framework applicable across larger regions may require a more process-based understanding of how topography influences tree cover through key mechanisms such as hydrology and fire. Mattos and others, (2023) demonstrate the potential of this approach by showing that topographic effects on soil hydrology explain forest–savanna co-occurrence in the Amazon. The potential role of local heterogeneities in transition zones has also been highlighted by modelling studies (Bastiaansen and others, 2022; Banerjee and others, 2026), but a more systematic understanding of varying topographic effects is needed to integrate these insights with realistic representations of topography. Advancing our understanding of forest–savanna transitions will therefore require explicitly incorporating topographic features into models and data studies, while allowing their effects on tree cover to vary spatially.

Our study area in Central Africa spans a large spatial extent and encompasses a diverse range of topographic conditions. We see no reason to assume that topography, or its effect on tree cover, would be fundamentally different in other tropical regions and therefore hypothesize that our findings may apply across the global tropics. Topography can be an important driver of tree cover anywhere, but as in our study area, its specific role will vary from region to region, partly depending on climatic conditions. Moreover, some of our findings may extend beyond the tropics, as mosaics of open and closed vegetation cover also occur in other climatic zones. For example, the extensive temperate forest–grassland mosaic spanning from central Europe to eastern Asia is governed by ecological dynamics similar to those maintaining tropical forest–savanna boundaries and is strongly influenced by topography (Erdős and others, 2022).

Here, we focused exclusively on topography as a driver of tree cover, motivated by its role as a heterogeneous template on which ecosystems develop. We can therefore only speculate on the mechanisms through which topography indirectly influences tree cover distributions in mosaic landscapes. Future work could examine in more detail how water redistribution and fire dynamics interact with topography, although such analyses remain challenging due to uncertainties in fire and soil moisture products (Brennan and others, 2019; Han and others, 2023). Estimating water availability directly from topography also remains difficult (Maduako and others, 2017; Kopecký and others, 2021), which is another reason we restricted our analysis to purely topographic features. Studies that jointly consider water- and fire-related topographic effects across larger spatial scales will be crucial for improving our understanding of global forest–savanna dynamics and the importance of local environmental heterogeneity in transition zones. Ongoing advances in satellite technologies may make such assessments increasingly feasible (Brocca and others, 2024; Qi and others, 2024), enabling more precise evaluation of how local topography shapes tree cover in mosaic landscapes.

## Conclusions

Our findings highlight the significant role of topographic heterogeneity in shaping forest–savanna mosaics in Central Africa, while suggesting a more limited role of positive feedbacks in determining vegetation states. Nevertheless, local forest–savanna distributions remain partly unexplained, meaning that a complete understanding of mosaic landscapes requires consideration of topographic features as well as fire and other potential drivers of tree cover. Local elevations relative to 50 m, 500 m and 5 km radii were the most important predictors of tree cover, although their importance and the direction of their effects varied substantially across space. This variation likely reflects how the influence of topographic mechanisms related to water availability and fire spread may vary depending on the local topographic characteristics and climatic conditions. Such variability complicates the generalization of topographic effects on tree cover and emphasizes the need to incorporate local processes and region-specific parameterizations when modelling tree cover across larger spatial scales. Understanding the role of topography as a driver of forest–savanna mosaics is especially important, as mosaic landscapes may buffer and smoothen transitions between forest and savanna ecosystems under changing environmental conditions.

## Supplementary Information

Below is the link to the electronic supplementary material.

Supplementary file1 (DOCX 1725 KB)

## Data Availability

The data and scripts that support the findings of this study are available at 10.5281/zenodo.17592399. These data were derived from the following resources available in the public domain: - FABDEM ver. 1.2, https://data.bris.ac.uk/data/dataset/s5hqmjcdj8yo2ibzi9b4ew3sn- Global Forest Change ver. 1.11, https://storage.googleapis.com/earthenginepartners-hansen/GFC-2023-v1.11/download.html- Global Land Cover 2000, https://forobs.jrc.ec.europa.eu/glc2000/data- WorldClim ver. 2.1, https://www.worldclim.org/data/worldclim21.html.
