# Supplementary material for: Topography is a Major Determinant of Forest–Savanna Distributions in Mosaic Landscapes in Central Africa
Source: Ecosystems. 2026 May 27;29(3):51. doi: 10.1007/s10021-026-01070-2 (PMC13216132; doi:10.1007/s10021-026-01070-2)
Supplement: Supplementary file 1 — Supplementary file1 (DOCX 1725 KB) [file 10021_2026_1070_MOESM1_ESM.docx]

Topography is a major determinant of forest–savanna distributions in mosaic landscapes in Central Africa

**Supporting information appendix S1: Figures**


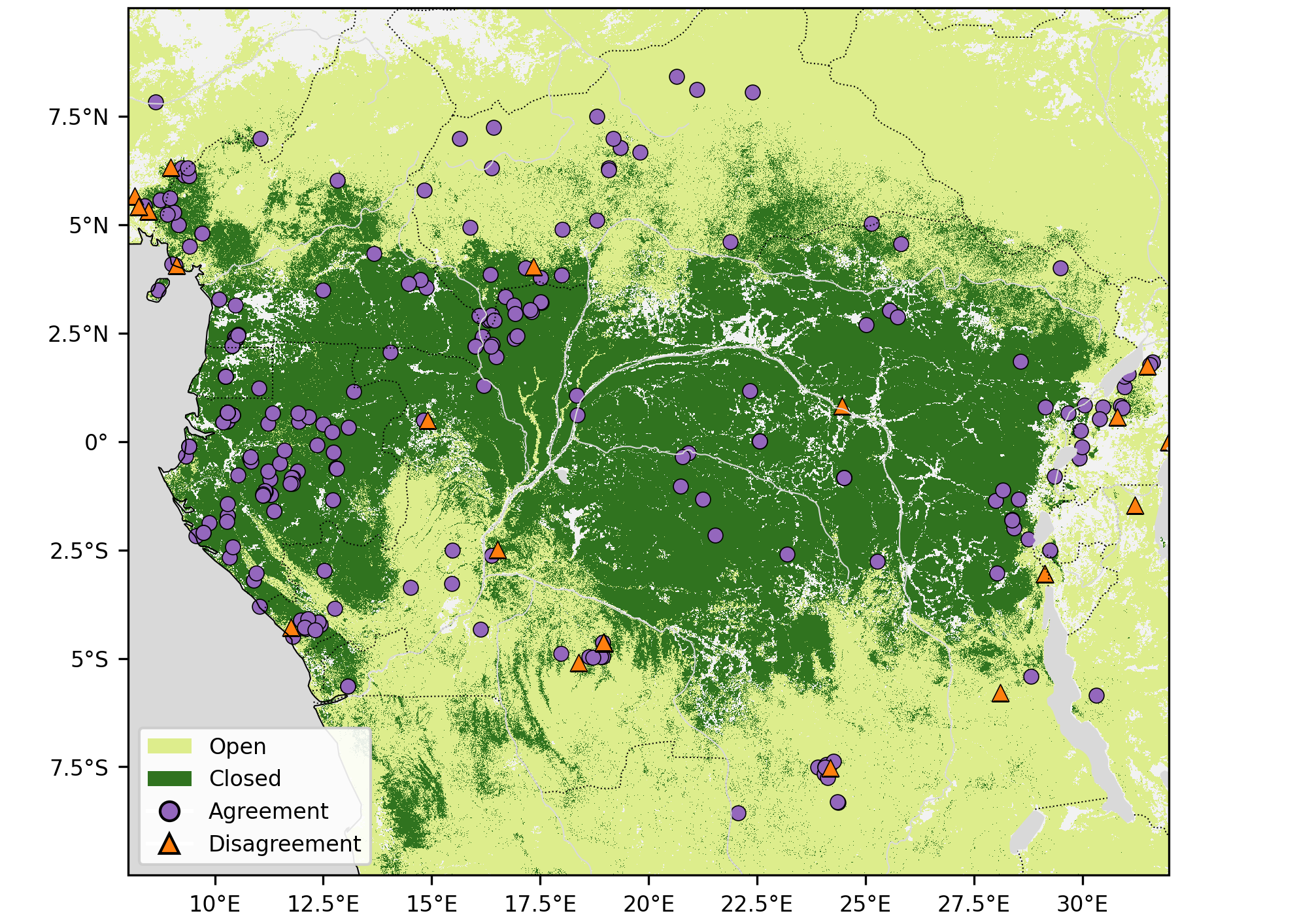


*Figure S1.1: Map of open (<65%) and closed (≥65%) tree cover in Central Africa at 30 m resolution for the year 2000, based on the Global Forest Change (GFC) ver. 1.11 (2000-2023) product (Hansen and others 2013). The Global Land Cover 2000 product (GLC2000, Bartholomé & Belward, 2005) was used to mask out pixels covered by surface water (codes 20-21) and pixels that experienced extensive human impact (codes 16-18, 22). Point data represent agreement (open = savanna, closed = forest) and disagreement between GFC-derived tree cover classes and field-based forest and savanna sites from Aleman and others (2020). Sites located on class boundaries (i.e., exactly between open and closed pixels) were excluded. Overall, agreement was found for 90% of the sites.*


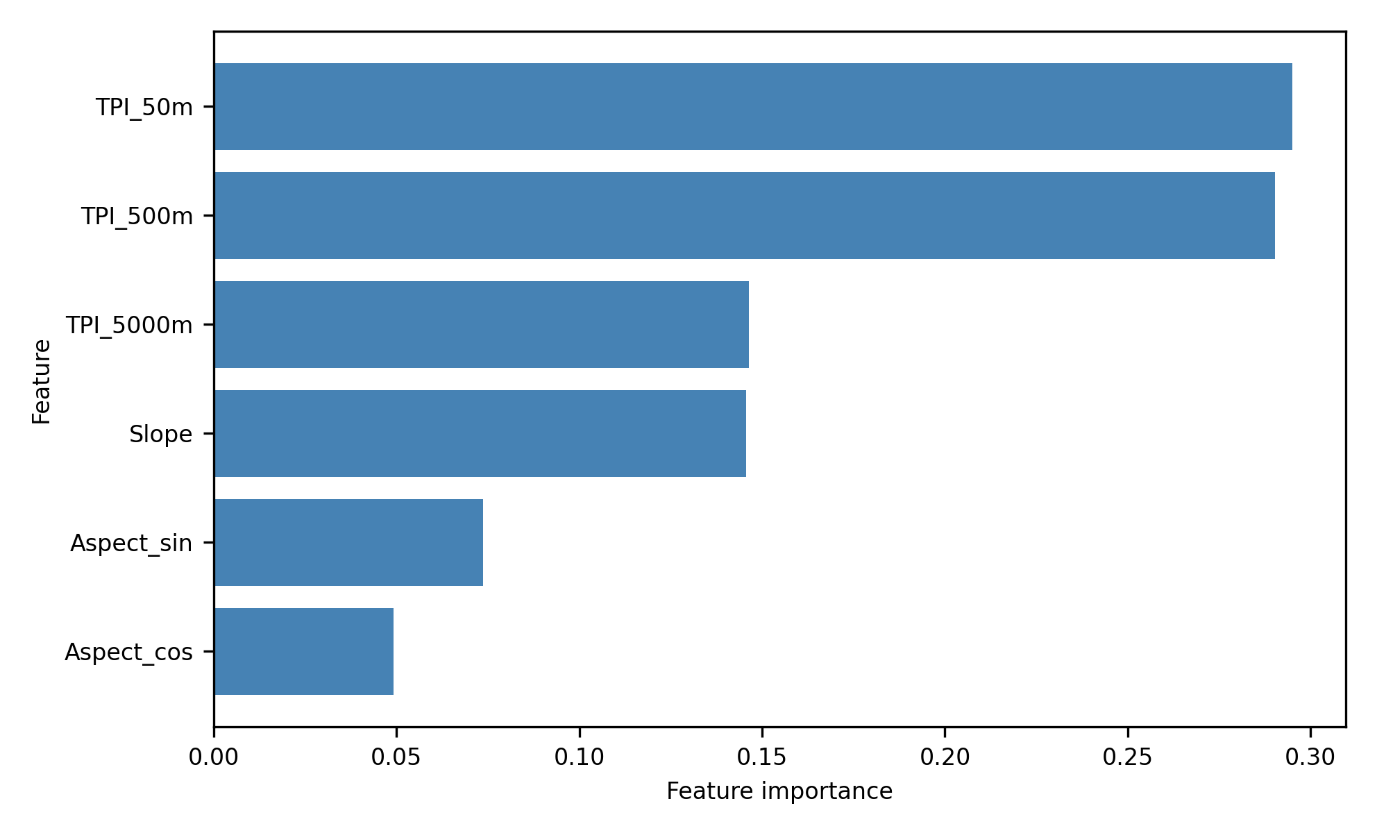


Figure S1.2: Feature importance of topographic variables in a regional random forest model predicting pixel type (open vs. closed tree cover) across all 0.1° x 0.1° mosaic landscapes in Central Africa. TPI_50m, TPI_500m, and TPI_5000 represent the topographic position index (local elevation) relative to neighbourhoods of 50, 500, and 5000 m. Slope indicates the slope angle, while Aspect_sin and Aspect_cos represent the sine and cosine of the slope aspect, corresponding to the eastness and northness of the slope orientation.


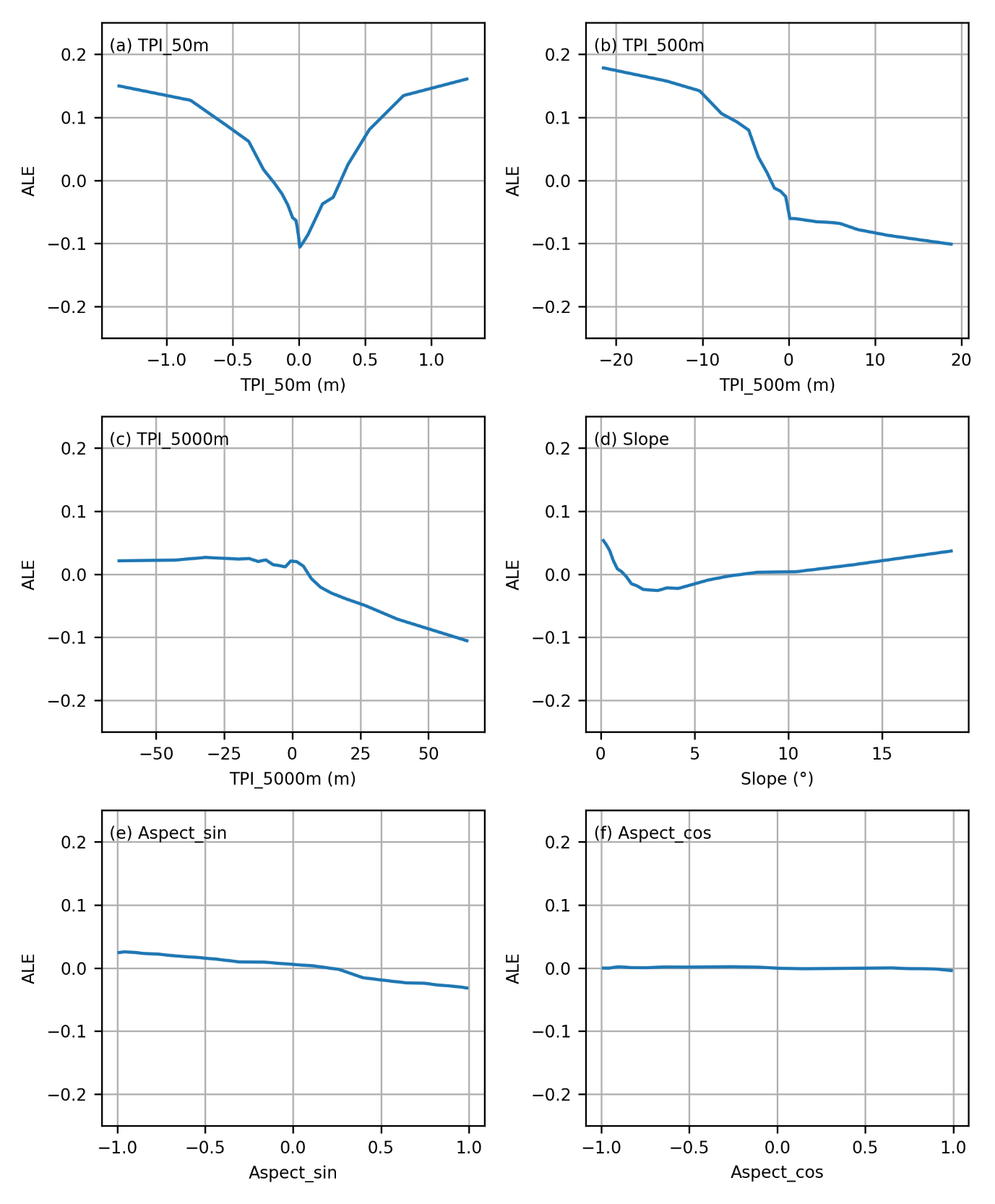


Figure S1.3: Accumulated Local Effect (ALE) plots showing the effect direction of topographic features in a regional random forest model predicting pixel type (open vs. closed tree cover) across all 0.1° x 0.1° mosaic landscapes in Central Africa. ALE values express the increase or decrease in the likelihood of the model predicting closed tree cover. TPI_50m, TPI_500m, and TPI_5000 represent the topographic position index (local elevation) relative to neighbourhoods of 50, 500, and 5000 m. Slope indicates the slope angle, while Aspect_sin and Aspect_cos represent the sine and cosine of the slope aspect, corresponding to the eastness and northness of the slope orientation.


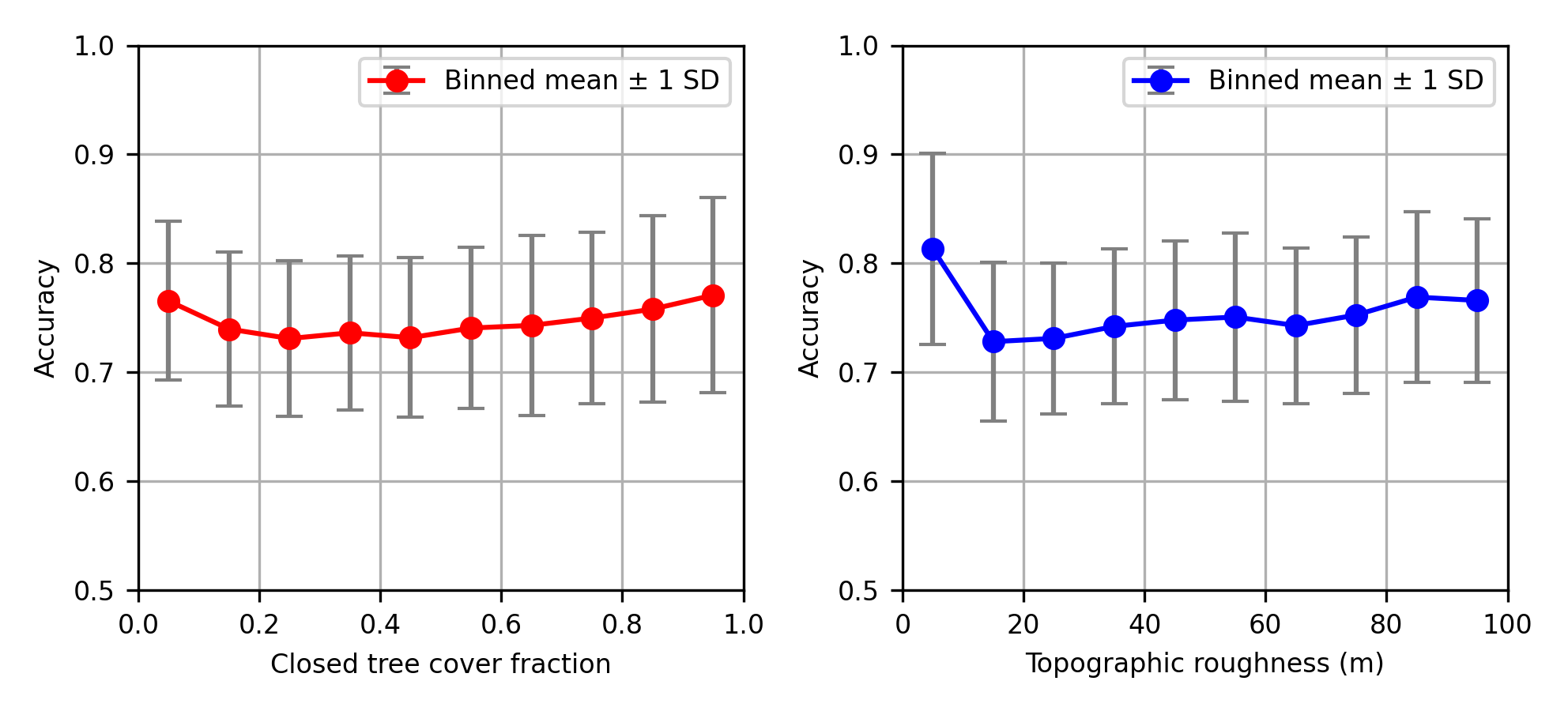


Figure S1.4: Dependence of model accuracy on landscape composition (a) and topographic roughness (b) for local random forest models predicting pixel type (open vs. closed tree cover) based on topographic variables. Local models were trained and evaluated separately for each 0.1° x 0.1° mosaic landscape. Accuracy is expressed as the proportion of model predictions that were correct.


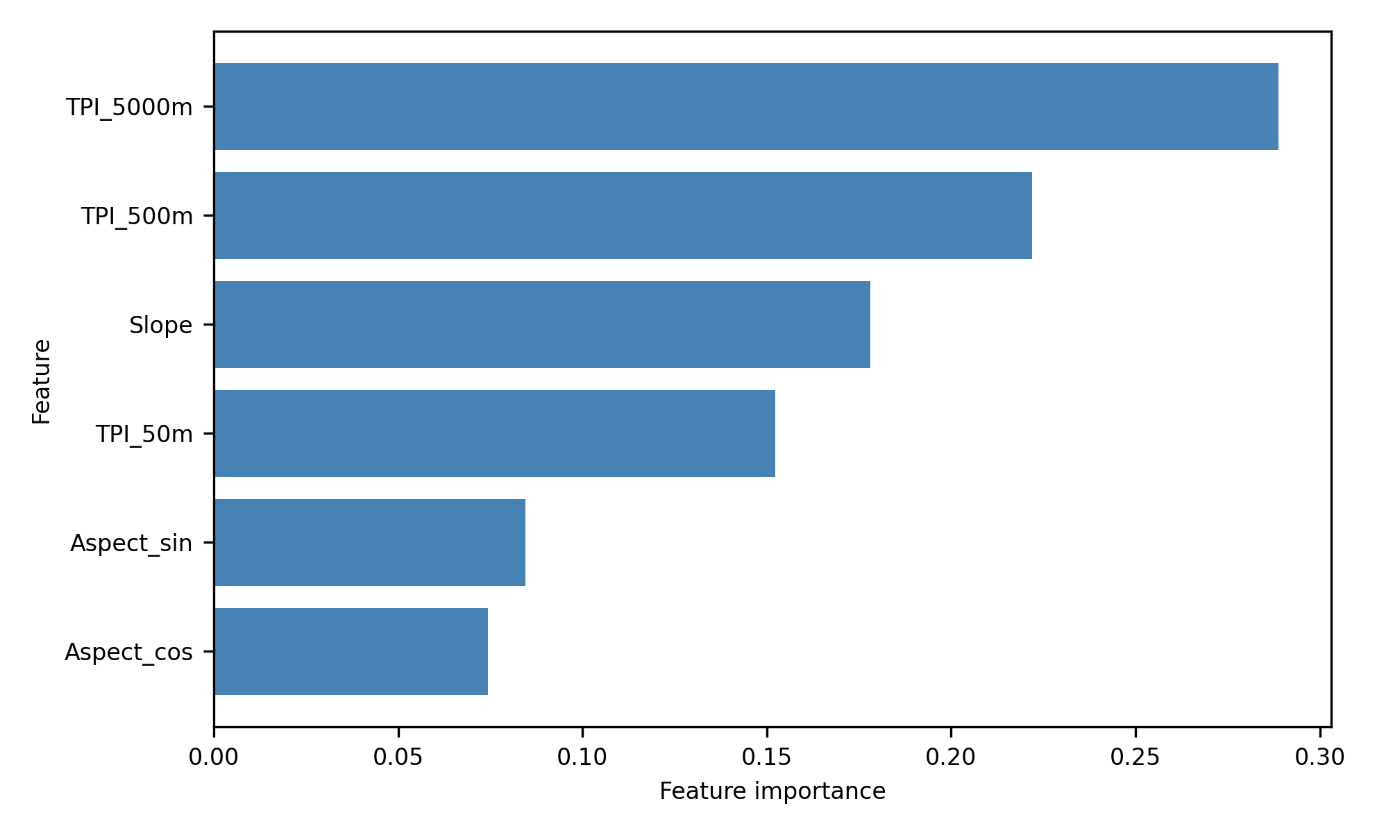


Figure S1.5: Average feature importance across local models predicting tree cover based on topographic variables. Local models were trained and evaluated separately for each 0.1° x 0.1° mosaic landscape. TPI_50m, TPI_500m, and TPI_5000 represent the topographic position index (local elevation) relative to neighbourhoods of 50, 500, and 5000 m. Slope indicates the slope angle, while Aspect_sin and Aspect_cos represent the sine and cosine of the slope aspect, corresponding to the eastness and northness of the slope orientation.


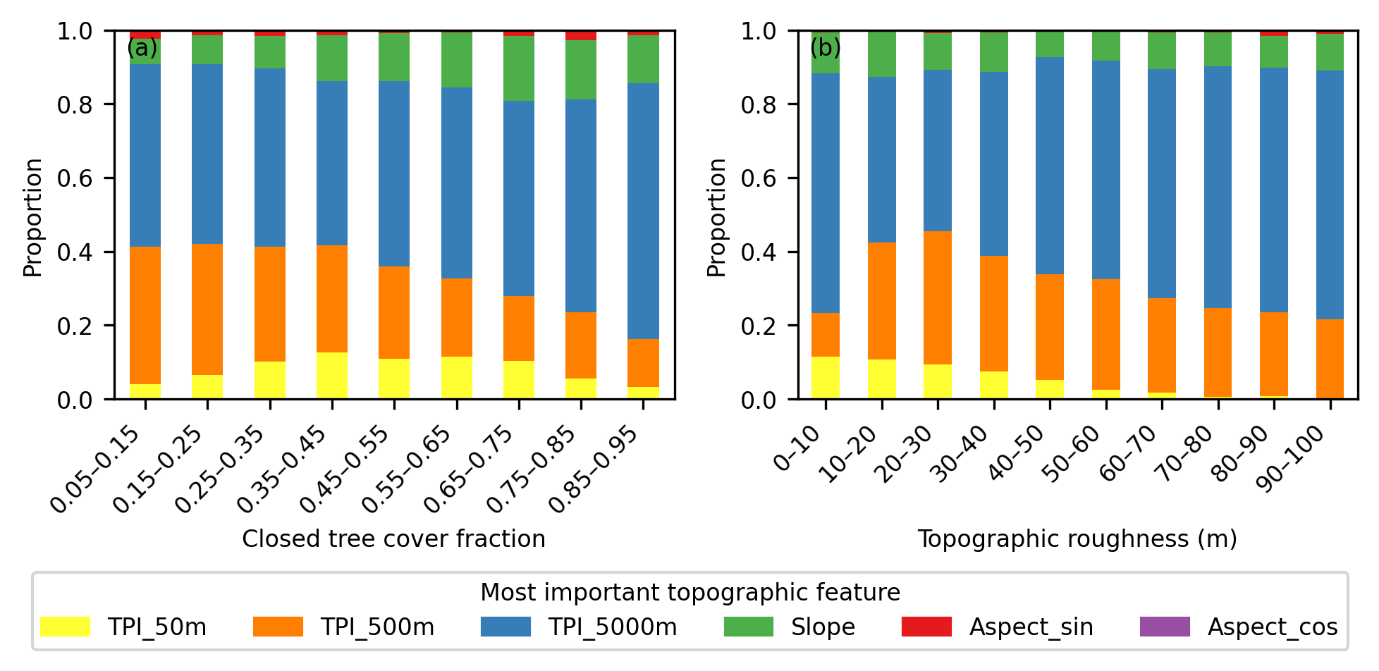


Figure S1.6: Dependence of feature importance on landscape composition (a) and topographic roughness (b) for local random forest models predicting pixel type (open vs. closed tree cover) based on topographic variables. Local models were trained and evaluated separately for each 0.1° x 0.1° mosaic landscape. TPI_50m, TPI_500m, and TPI_5000 represent the topographic position index (local elevation) relative to neighbourhoods of 50, 500, and 5000 m. Slope indicates the slope angle, while Aspect_sin and Aspect_cos represent the sine and cosine of the slope aspect, corresponding to the eastness and northness of the slope orientation.


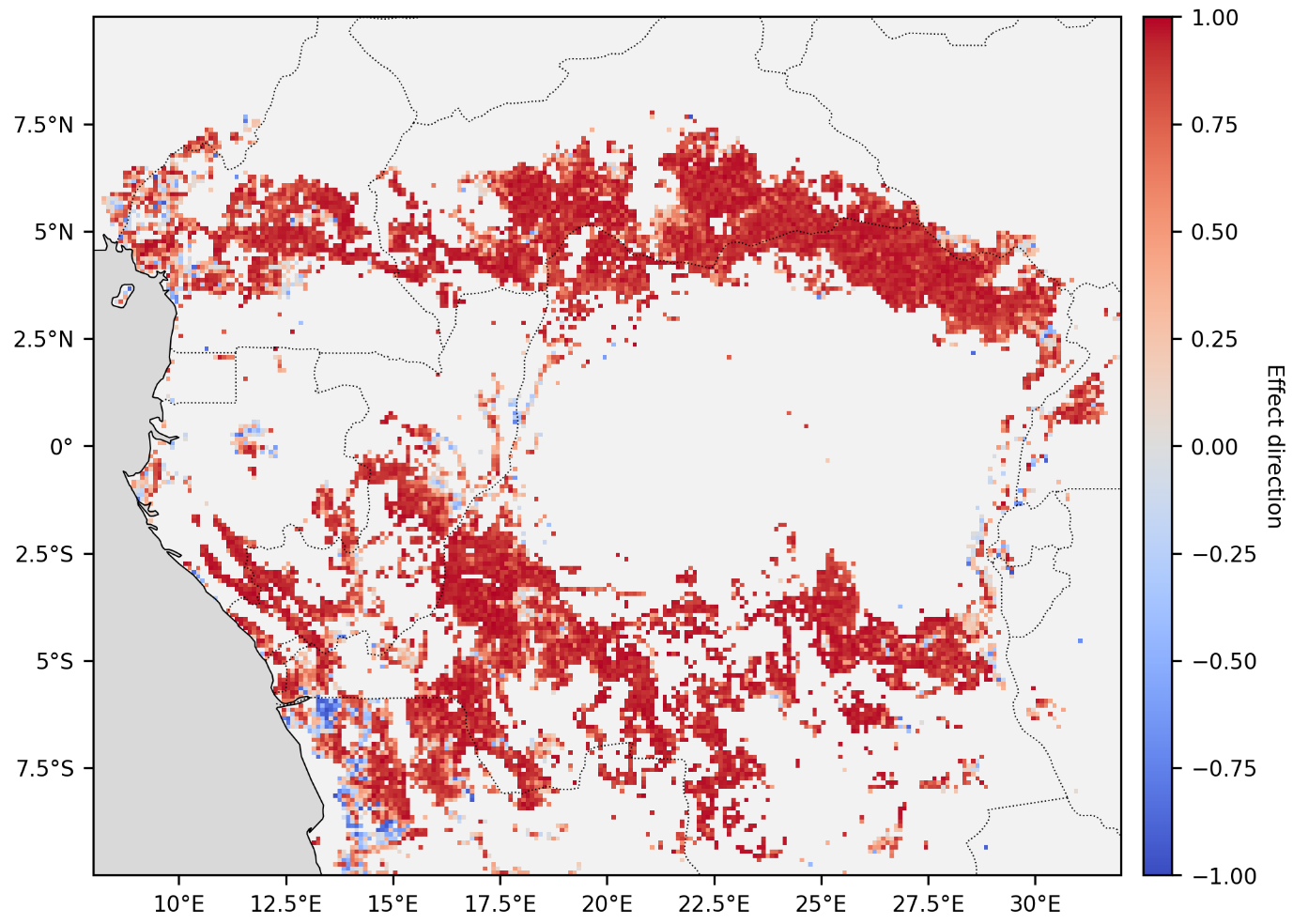


Figure S1.7: Effect direction of the absolute value of TPI_50m on the likelihood of a local model predicting closed tree cover, quantified as the Spearman correlation of the Accumulated Local Effect (ALE) plot. Positive values indicate that higher feature values increase the likelihood of predicting closed tree cover, while negative values indicate that higher feature values decrease this likelihood. The magnitude reflects the monotonicity of this trend within a landscape. Local random forest models predicting pixel type (open vs. closed tree cover) based on topographic variables were trained and evaluated separately for each 0.1° x 0.1° mosaic landscape. TPI_50m represents the topographic position index (local elevation) relative to a neighbourhood of 50 m.


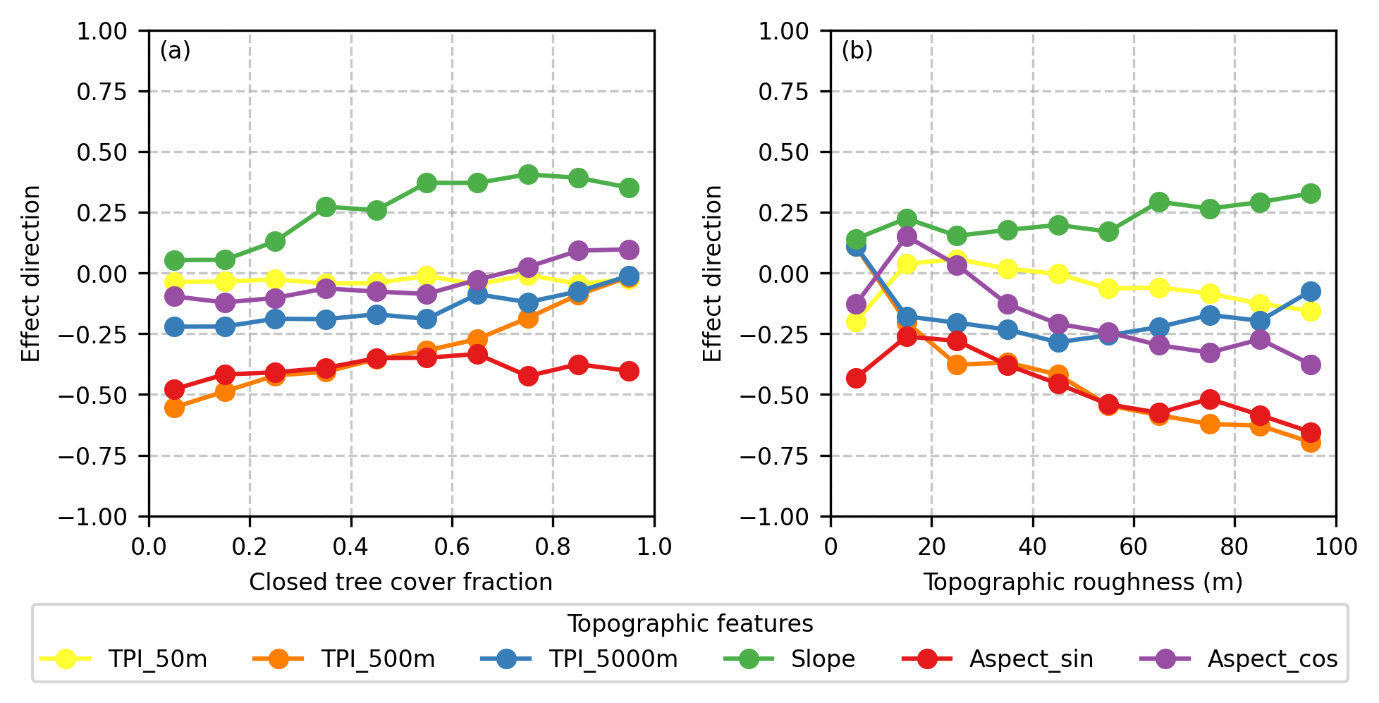


Figure S1.8: Dependence of feature effect directions on landscape composition (a) and topographic roughness (b) for local random forest models predicting pixel type (open vs. closed tree cover) based on topographic variables. Local models were trained and evaluated separately for each 0.1° x 0.1° mosaic landscape. Effect direction is quantified as the Spearman correlation of the Accumulated Local Effect (ALE) plot for each feature. Positive values indicate that higher feature values increase the likelihood of predicting closed tree cover, while negative values indicate that higher feature values decrease this likelihood. The magnitude reflects the monotonicity of this trend within a landscape. TPI_50m, TPI_500m, and TPI_5000 represent the topographic position index (local elevation) relative to neighbourhoods of 50, 500, and 5000 m. Slope indicates the slope angle, while Aspect_sin and Aspect_cos represent the sine and cosine of the slope aspect, corresponding to the eastness and northness of the slope orientation.


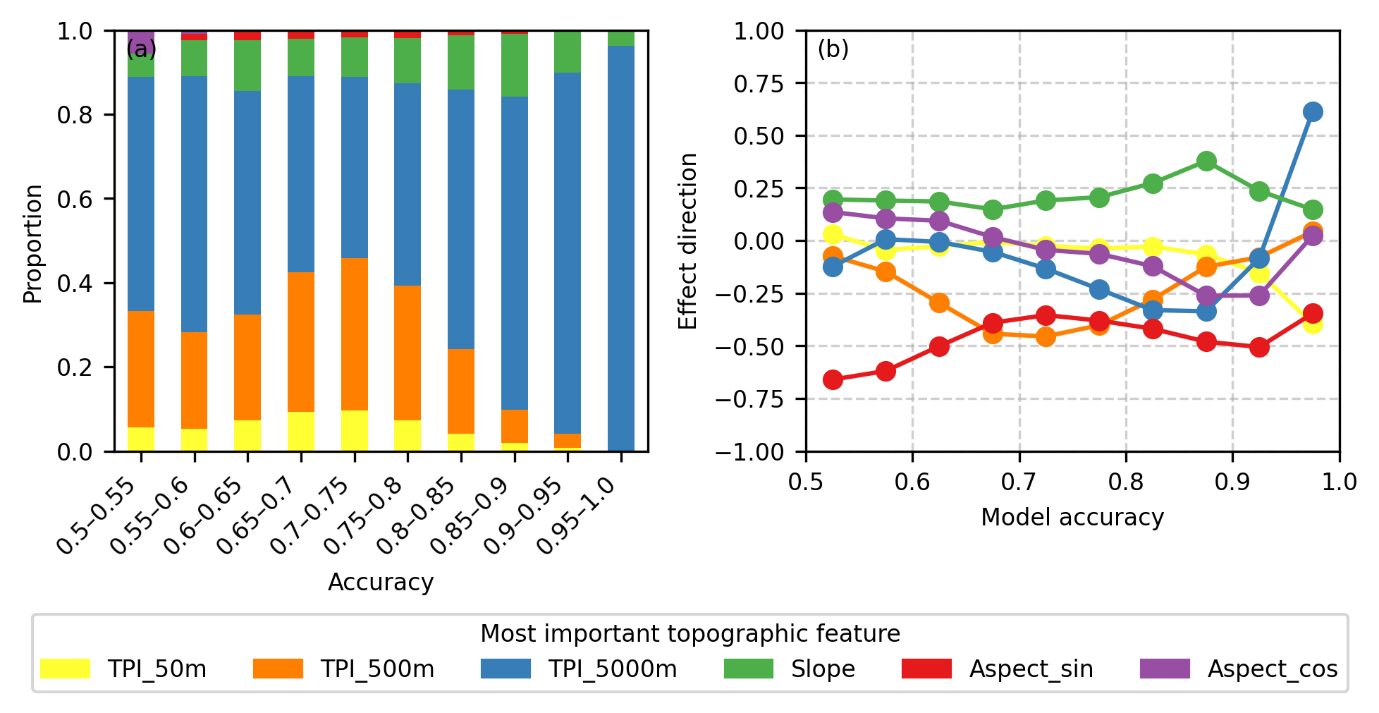


Figure S1.9: Dependence of feature importance (a) and effect direction (b) on model accuracy for local random forest models predicting pixel type (open vs. closed tree cover) based on topographic variables. Local models were trained and evaluated separately for each 0.1° x 0.1° mosaic landscape. TPI_50m, TPI_500m, and TPI_5000 represent the topographic position index (local elevation) relative to neighbourhoods of 50, 500, and 5000 m. Slope indicates the slope angle, while Aspect_sin and Aspect_cos represent the sine and cosine of the slope aspect, corresponding to the eastness and northness of the slope orientation. Effect direction is quantified as the Spearman correlation of the Accumulated Local Effect (ALE) plot for each feature. Positive values indicate that higher feature values increase the likelihood of predicting closed tree cover, while negative values indicate that higher feature values decrease this likelihood. The magnitude reflects the monotonicity of this trend within a landscape.
